# Mechanisms of chronic alcohol exposure-induced aggressiveness in cellular model of HCC and recovery after alcohol withdrawal

**DOI:** 10.1007/s00018-022-04387-y

**Published:** 2022-06-17

**Authors:** Constance Marié, Grégory Fouquet, Anoïsia Courtois, Rabbind Singh Amrathlal, Nicolas Jankovsky, Hakim Ouled-Haddou, Riad Tebbakha, Hicham Bouhlal, Éric Nguyen-Khac, Mickaël Naassila, Ingrid Marcq

**Affiliations:** 1 Groupe de Recherche Sur L’Alcool et Les Pharmacodépendances INSERM UMR1247, Centre Universitaire de Recherche en Santé CURS, Université de Picardie Jules Verne, Avenue Laennec, 80051 Amiens, France; 2 Department of Hepatogastroenterology, Centre Hospitalier Universitaire Sud, Amiens, France; 3 Centre for Genomics, Jiwaji University, Gwalior, 474011 India; 4 Laboratoire HEMATIM EA4666, Centre Universitaire de Recherche en Santé CURS, Université de Picardie Jules Verne, Amiens, France; 5 Service Anatomie Pathologie-Tumorothèque de Picardie, Centre Hospitalier Universitaire Nord, Amiens, France

**Keywords:** Liver cancer, Alcohol, Cancerous stem cell, Withdrawal

## Abstract

Alcohol-related liver disease is the most prevalent chronic liver disease worldwide, accounting for 30% of hepatocellular carcinoma (HCC) cases and HCC-specific deaths. However, the knowledge on mechanisms by which alcohol consumption leads to cancer progression and its aggressiveness is limited. Better understanding of the clinical features and the mechanisms of alcohol-induced HCC are of critical importance for prevention and the development of novel treatments. Early stage Huh-7 and advanced SNU449 liver cancer cell lines were subjected to chronic alcohol exposure (CAE), at different doses for 6 months followed by 1-month alcohol withdrawal period. ADH activity and ALDH expression were much lower in SNU449 compared with Huh-7 cells and at the 270 mM dose, CAE decreased cell viability by about 50% and 80%, respectively, in Huh-7 and SNU449 cells but induced mortality only in Huh-7 cells. Thus, Huh-7 may be more vulnerable to ethanol toxicity because of the higher levels of acetaldehyde. CAE induced a dose-dependent increase in cell migration and invasion and also in the expression of cancer stem cells markers (CD133, CD44, CD90). CAE in Huh-7 cells selectively activated ERK1/2 and inhibited GSK3β signaling pathways. Most of the changes induced by CAE were reversed after alcohol withdrawal. Interestingly, we confirmed the increase in CD133 mRNA levels in the tumoral tissue of patients with ethanol-related HCC compared to other HCC etiologies. Our results may explain the benefits observed in epidemiological studies showing a significant increase of overall survival in abstinent compared with non-abstinent patients.

**Supplementary Information:**

The online version contains supplementary material available at 10.1007/s00018-022-04387-y.

## Introduction

Ratio of alcohol consumption to all other etiologies of hepatocellular carcinoma (HCC) varies according to the country and area. Alcohol use is reported to be responsible for 30.4% of HCC globally [1]. HCC is the fourth leading cause of cancer-related mortality worldwide while its incidence is ranked sixth [2]. Only very few patients who are diagnosed at an early stage may benefit from cancer resection or liver transplantation. Majority of HCC cases are diagnosed in advanced stage of the disease and present a 3-year survival lower than 10% [3, 4]. Sorafenib, one of the first-line treatment for advanced HCC, is a mere palliative targeted therapy which improves overall survival by only 3 months [5, 6]. One of the main etiologies of HCC, more particularly in Europe, is alcohol consumption [7]. Alcohol consumption is the fifth leading cause of premature death, resulting in 2.84 million deaths globally in 2017 [2]. Apart from HCC, alcohol consumption can also lead to the development of diseases such as ARLD (alcohol-related liver disease) [8, 9]. ARLD is defined by a broad spectrum of clinical and biological liver states whose HCC remains one of the most critical pathologies [10, 11].

Alcohol is classified by the International Agency for Research on Cancer (IARC) as group 1 human carcinogens. Upon consumption, alcohol is absorbed in the small intestine and 90% is metabolized by the liver in three ways. The major alcohol metabolism pathway is through oxidation by alcohol dehydrogenase (ADH). This reaction results in the production of acetaldehyde, a carcinogenic compound, which is enzymatically reduced to acetate by aldehyde dehydrogenase (ALDH) [12]. The activity of ADH seems to be crucial in alcohol-related cancers such as HCC [13].

Alcohol-related HCC is diagnosed outside surveillance leading to a worse diagnostic and overall survival than other etiologies [14]. At an advanced stage, HCC presents an aggressive phenotype. Numerous mechanisms such as acquisition of cancer stem cells (CSCs), a subpopulation characterized by self-renewal and differentiation abilities can explain HCC aggressiveness. Indeed, enrichment of CSCs, contributes to cancer development and metastasis [15]. These cells are phenotypically positive for markers such as CD133, CD44, CD73, CD90, and CD24 and present a high cell migration and invasive potential [16]. Huh-7 cells in culture abundantly express CD133 on their surface and Suetsugu et *al.* have shown that CD133^+^ cells, isolated from culture of Huh-7, have higher proliferative and tumorigenic capacity in comparison with CD133^−^ cells isolated from the same batch of cells [17]. Moreover, CD133^+^CD44^+^ subpopulation was demonstrated, in vivo, more tumorigenic than the CD133^+^CD44^*−*^ subpopulation [18]. The expression of CD73, another CSCs marker, is implicated in the sphere-forming in HCC cells with an increase of metastatic ability [19]. Other studies determined the part of CD90 and CD24 [16]. Interestingly, in vivo study, demonstrated the induction of CD133^+^ subpopulation after ethanol-fed exposure in Diethylnitrosamine (DEN) HCC model [20, 21].

To elucidate how ethanol exposure may contribute to cancer progression, many studies have used cell cultures exposed to ethanol. The induction of aggressiveness by ethanol exposure depends on the cell type. For example, 16–72 h of exposure to 0–44 mM ethanol is sufficient to induce aggressiveness in colorectal cancer cells [22, 23], but a long-term ethanol exposure is needed in breast cancer cells which require longer exposure (up to 2 months) [24, 25]. In HCC, chronic ethanol exposure between 4 and 9 days has been suggested to be sufficient [26, 27] in HepG2 cell line which is deficient in ADH activity [28]. Thus, the HepG2 cellular model was unsuitable to capture the role of the ethanol metabolite acetaldehyde, an important carcinogen. Mechanisms underlying the role of ethanol exposure on HCC development and aggressiveness need to be further investigated.

Several studies demonstrate that aberrant activation of several molecules in various signaling pathways controlling cell cycle, proliferation, differentiation, cell survival, and apoptosis. Indeed, upregulation of Wnt/GSK3β/β-catenin pathway, PI3K/AKT/mTOR pathway and Ras/Raf/MAPK pathways conduce to HCC progression [29, 30].

Here, we explored the pathophysiologic mechanisms underlying the impact of CAE on the development of HCC. We first used Huh-7 and SNU449 cell lines to characterize the duration and the dose of ethanol and established that a very chronic exposure is required (at least 6 months). In this model, we also carried out a withdrawal (WD) period to mimic the impact of abstinence on HCC. We show that CAE induced an acquisition of aggressive phenotype in Huh-7 cell line and this alteration was reversed by withdrawal. In Huh-7 cell line, CAE led to an increase of migration, invasive potentials and expression of CD133, CD44, CD90, and CD24. Along with modulation of CSC markers, we further show that CAE activates AKT, ERK1/2 and inhibits GSK3β in early grade HCC cells and that their expression levels were restored to the level of the control group at 1 month of withdrawal. In cohort of HCC patients, our results showed a higher expression of the CSC marker CD133 in HCC patients with alcohol use disorder compared to HCC of other etiologies. Our data shed light on the pathophysiology of alcohol-related HCC and demonstrate a beneficial effect of alcohol withdrawal on aggressive mechanisms induced by CAE in HCC cells.

## Materials and methods

### Cell lines and culture conditions

Early grade HCC cell line Huh-7 was maintained in Dulbecco’s Modified Eagle’s Medium while SNU449, a cell line of advanced grade hepatocellular carcinoma, in RPMI1640 medium. SNU449 are cells of grade II–III/IV of human HCC (American Type Culture Collection reference CRL-2234). Cell culture medium was supplemented with 10% foetal calf serum, L-glutamine (2 mM), penicillin (100 U/mL), and streptomycin (100 µg/mL) (Dutscher, Issy-les-Moulineaux, France). Both cell lines were grown in a humidified incubator with 5% CO2 at 37 °C and routinely tested for mycoplasma contamination.

### Patient samples

In protocol PI2022_843_0016, sixteen (*n* = 16) periTumoral (pT) tissues and fifty-one (*n* = 51) Tumoral (T) tissues were obtained from HCC patients undergoing surgical resection at Amiens University Hospital (Amiens, France) stored at Tumorothèque of Picardie (ISO 9001–2015). Among these, twenty-nine (*n* = 29) tumoral samples were obtained from patients with alcoholic HCC and twenty-two (*n* = 22) from patients with HCC of other etiology. Total mRNA was extracted using specific kits and used to test CD133 and CD90 expression.

### Chronic alcohol exposure (CAE) and withdrawal

**Fig. 1:**
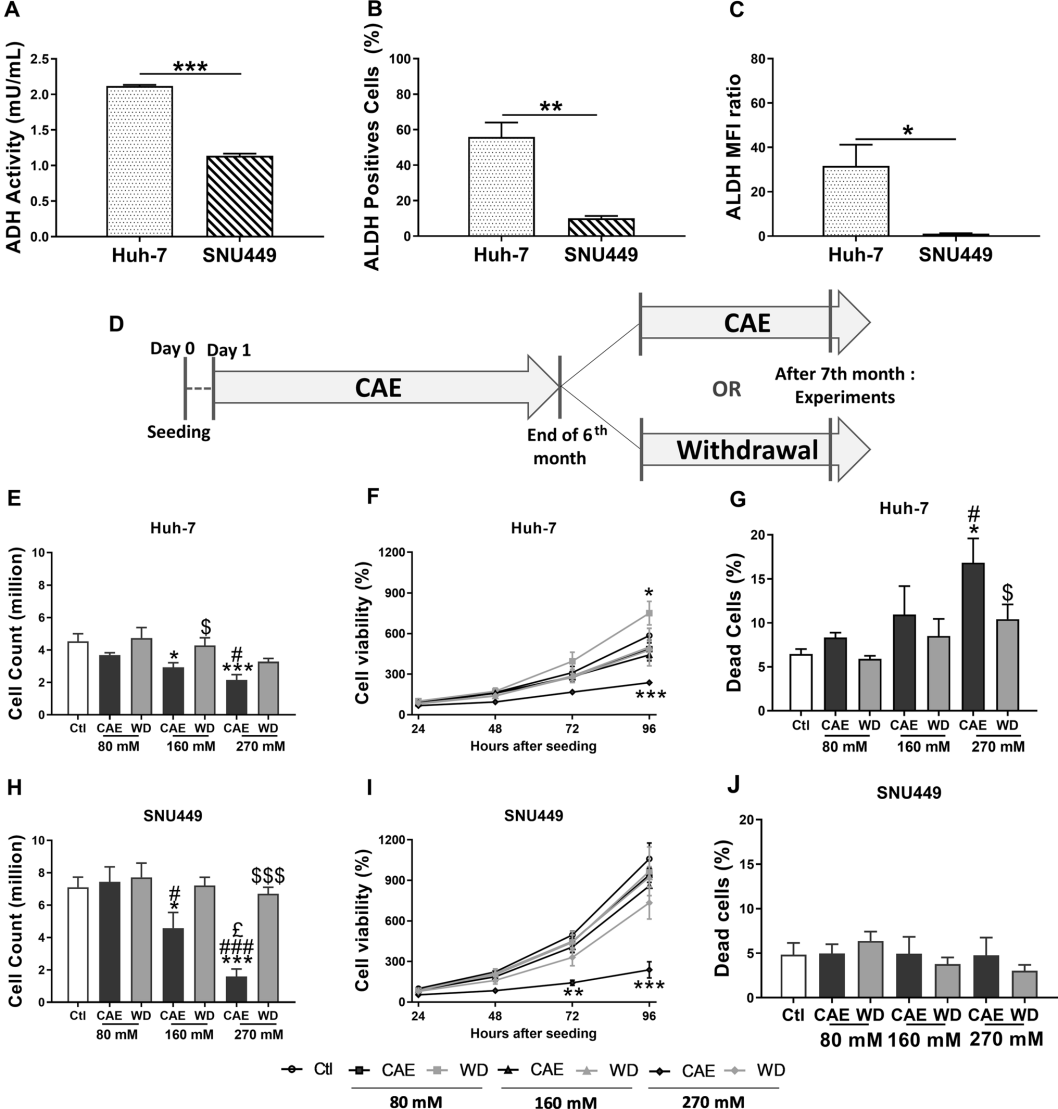
CAE protocol on HCC cell lines, alcohol metabolism, cell viability, and benefits of withdrawal. Cells were cultured in the presence of several doses of alcohol (80 mM, 160 mM or 270 mM) during 6 months. At the end of the 6th month, cells were splitted in two parts: one which continued its alcohol exposition, and the second was subject to a withdrawal during 1 month (**A**). Alcohol DeHydrogenase (ADH) activity was studied in Huh-7 and SNU449 cells. Results represented mean ± SEM of three independent experiments (*N* = 3, ***p < 0.001) (**B**). Number of ALdehyde DeHydrogenase (ALDH) positives cells (**C**) and relative mean fluorescence intensity (**D**) were determined using flow cytometry in Huh-7 et SNU449 cells. Results represented mean ± SEM of three independent experiments (*N* = 3, *p < 0.05, **p < 0.01). Cell counting was realized using trypan blue. Results represented mean ± SEM of five independent experiments (*N* = 5, *p < 0.05 vs Ctl, ***p < 0.001 vs Ctl, ^#^*p* < 0.05 vs 80 mM, ^###^*p* < 0.001 vs 80 mM, ^£^*p* < 0.05 vs 160 mM, ^$^*p* < 0.05 vs respective CAE condition, ^$$$^*p* < 0.001 vs respective CAE condition) (**E** and** H**). To determine cell viability, MTT assay was performed 24 h, 48 h, 72 h, and 96 h after seeding. Results represented mean ± SEM of four independent experiments (*N* = 4, *p < 0.05 vs Ctl, ***p < 0.001 vs Ctl) (**F** and **I**). Cell mortality was determined using propidium iodide in flow cytometry. Results represented mean ± SEM of three independent experiments (*N* = 3, ^#^*p* < 0.05 vs 80 mM, ^$^*p* < 0.05 vs respective CAE condition) (**G** and **J**)

To model development of HCC upon several years of excessive alcohol consumption in humans, HCC cell lines routinely cultured in media lacking ethanol were grown in media containing ethanol for at least 6 months as reported in our earlier pilot studies. For experimental purposes cells grown in media containing ethanol were considered Chronic alcohol exposure (CAE) while cells grown in media devoid of alcohol for 1-month mimic alcohol withdrawal (Fig. 1D). In our conditions, 24 h after cell seeding, the required amount of alcohol is added to the culture medium. We measured ethanol evaporation in our culture conditions and to offset loss of ethanol due to evaporation, 25% of the dose was added to the culture flask everyday.

### Antibodies

Antibodies used for immunofluorescence, flow cytometry or western blotting are listed in Suppl table 1.

### Statistical analysis

Data obtained for cell lines Huh-7 and SNU449 were compared using Student’s t test if the values were normally distributed otherwise non-parametric Mann–Whitney test was applied. To compare the data obtained for CAE and WD conditions, two-way ANOVA following Holm–Sidak’s method was used. When variance equality was not respected, ANOVA on ranks was performed (non-parametric Kruskal–Wallis test). All the results are represented as mean ± SEM and the threshold for statistical significant was set to *p* < 0.05. All the statistical analyses were performed using SigmaPlot software (version 11.0, Systat Software Inc., San Jose, CA, USA).

Details of the other methods, materials, and reagents used including « Alcohol DeHydrogenase assays», « ALdehyde DeHydrogenase Assays», « Cell Cycle analysis», « Cell Viability assays», « Flow cytometry», « Immunofluorescence assays», « Matrix MetalloProteinases (MMP)’s global activity assays», « Migration and Invasion assays», « Mortality analysis», « RNA extraction, quantitative real time PCR», and « Western Blot analysis» are provided in the supplementary files.

## Results

### SNU449 cells are less sensitive to alcohol metabolism induced toxicity

First, we determined differences in ADH activity and ALDH expression between Huh-7 and SNU449 cell lines. We observed that advanced grade HCC cell line SNU449 displays a 1.86-fold lower ADH activity in comparison to Huh-7 cells, an early grade HCC cell line (Fig. 1A). We also compared the ALDH expression in both Huh-7 and SNU449 using flow cytometry and observed lower number of ALDH positive cells in SNU449. This observation was further confirmed by the lower mean fluorescence intensity (MFI) observed in the case of SNU449 cells (Fig. 1B and C). Cell lines were cultured according to the protocol described in Fig. 1D, showing a 1-month alcohol withdrawal period after CAE. Our results show that CAE and WD had no effect on the ADH activity profile of Huh-7 and SNU449 cells (Suppl Fig. 1A and 1B). When Huh-7 cells were exposed to alcohol, the number of ALDH positive cells as well MFI displayed an increasing trend in CAE that was absent after WD (Suppl Fig. 1C and 1E). Interestingly, when SNU449 cells underwent 270 mM alcohol treatment significant increase of ALDH positive cells and elevated MFI was observed with a recovery seen in conditions of WD. (Suppl Fig. 1D and 1F). Thus, it seems that the increase in ALDH expression seen in SNU449 exposed to 270 mM alcohol reflect a mechanism of tolerance for a quick elimination of the toxic molecule acetaldehyde. The tolerance may be a protective factor and could influence cell survival.

### Chronic alcohol exposure induces an aggressive phenotype while withdrawal reverses this effect

Trypan blue staining and counting of Huh-7 cells followed by alcohol exposure and WD revealed that CAE leads to the dose-dependent reduction of cell number. In comparison to untreated/control cells, the decrease of cell number was 18.40% and 52.54% at the 80 mM and 270 mM dose, respectively (Fig. 1E). Similarly, in the case of SNU449 cells, we observed a dose response in the effect of CAE on cell number with no effect at the 80 mM dose, a 35.49% reduction at the 160 mM dose and 77.60% reduction at the 270 mM dose (Fig. 1H). WD partially restored the cell number both in Huh-7 and SNU449.

To confirm the reduction in cell number by CAE observed by Trypan blue staining, cell viability assay was performed according to protocol mentioned in supplementary materials and methods. Cell viability was assessed by MTT assay at 24, 48, 72, 96 h time points. In Huh-7 cells we observed 59.54% decrease in viability in the 270 mM CAE condition (Fig. 1F). The reduction in cell viability at 270 mM CAE in SNU449 cells was 77.49% which was significantly higher in comparison to Huh-7 (Fig. 1I).

Cell mortality was also assessed by propidium iodide staining followed by flow cytometry. Figure 1G shows a dose-dependent increase in the dead cell count observed in Huh-7 cells after CAE. A partial recovery was observed after WD. Strikingly, CAE did not impact the cell mortality in SNU449 cells (Fig. 1J). We also tested the effect of CAE and WD on the cell cycle in Huh-7 and SNU449 cells and observed no effect (Suppl Fig. 2A and 2B).

Indeed, the cell count (Fig. 1H) and the MTT colorimetric assay (Fig. 1I) show a significant decrease in 270 mM CAE treated SNU449 cell line but dead cell did not increase significantly (Fig. 1J). In our cell cultures, we do not observe any cell death. The cells are adherent and alive but less numerous. The most probable hypothesis seems to be a slowing down of cell proliferation.

### Withdrawal reverses aggressive phenotype induced by alcohol in Huh-7 cell line

**Fig. 2:**
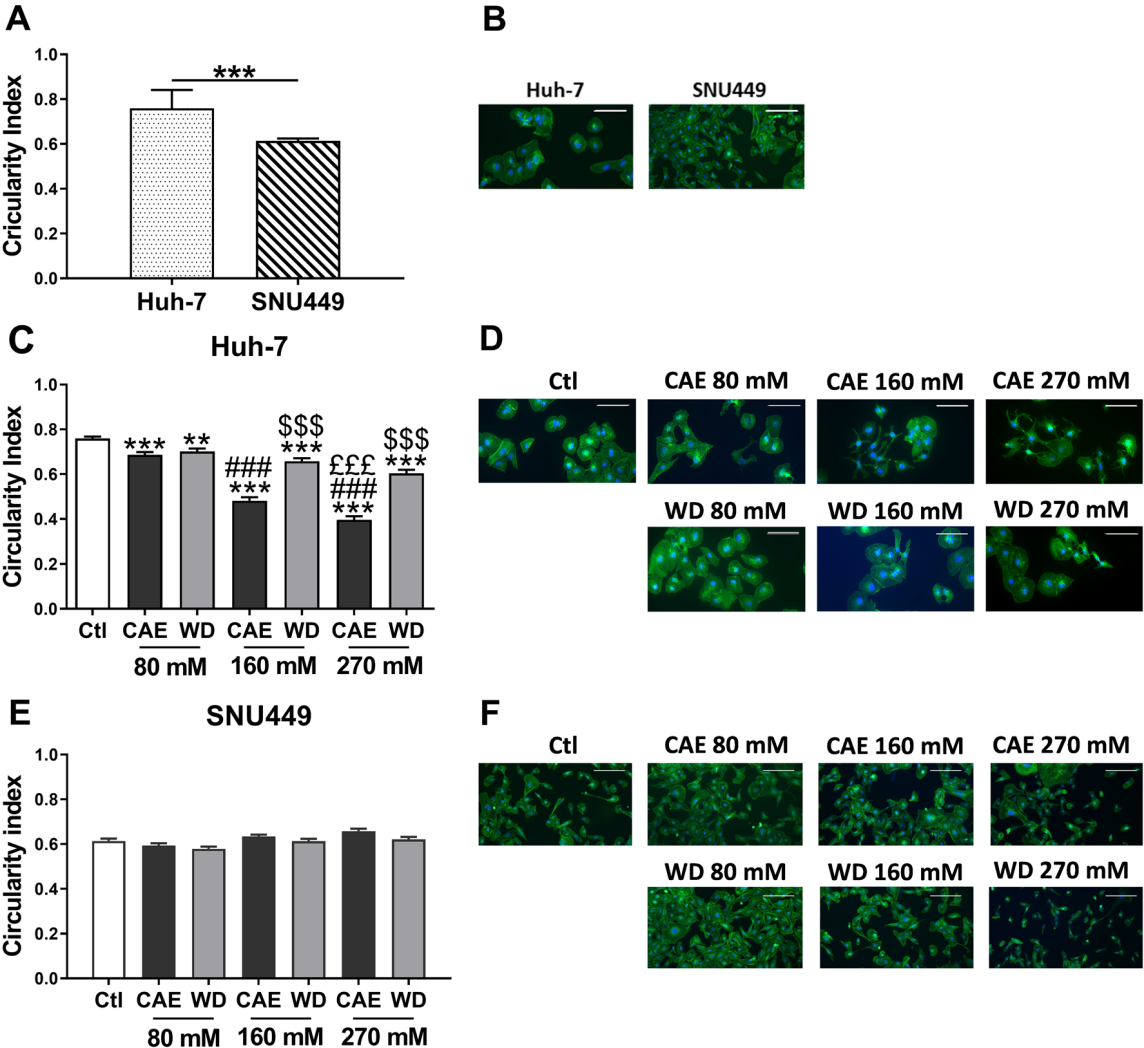
CAE induced modification of cell morphology reversed by withdrawal. To determine cell morphology, circularity index was analyzed using Image J software. Results represented mean ± SEM of three independent experiments (*N* = 3, *n* = 210, ***p < 0.001 vs Ctl, ^###^*p* < 0.001 vs 80 mM, ^£££^*p* < 0.001 vs 160 mM, ^$$$^*p* < 0.001 vs respective CAE condition) (**A**, **C**, and** E**). Morphology of Huh-7 and SNU449 cells were studied by immunofluorescence assay using phalloïdine conjugated with AlexaFluor488 (green). Nucleus was stained with DAPI (blue). Photographs represents one of the three independent experiment (*N* = 3) (**B**, **D**, and **F**)

Acquisition of aggressive phenotype in cancer cells can be reflected by a change in cell morphology in which circularity index serves as a quantifiable trait. Fluorescence microscopy was used to investigate the morphological changes in Huh-7 and SNU449 cell lines under CAE and WD conditions. Cells were stained with phalloidin conjugated with Alexafluor 488 to visualize cytoskeleton and DAPI to stain the nucleus. Circularity index of each cell line was determined under untreated conditions. We observed that Huh-7 cells presented a higher circulatory index than SNU449 (Fig. 2A and B). When Huh-7 cells underwent CAE, a significant dose-dependent decrease of circularity was observed: 9.21% in 80 mM, 36.84% in 160 mM, and 47.37% in 270 mM ethanol concentration. Interestingly, WD partially reversed the change in circularity index induced by CAE in Huh-7 cells. The shortfall in circularity index was 7.89% in WD 80 mM, 13.16% in WD 160 mM, and 21.05% in the case of WD 270 mM. Thus, the results presented here suggest that Huh-7, an early grade cancer cells acquire phenotype similar to that of aggressive cells upon exposure to alcohol with partial recovery when alcohol exposure is withdrawn (Fig. 2C and D ). However, it is noteworthy that CAE/WD did not have a significant impact on the circularity index of SNU449 cells (Fig. 2E and F). Thus, CAE can induce early grade cancer cells such as Huh-7 to acquire a phenotype resembling SNU449, an advanced cancer cell while WD can be beneficial as it can reverse the CAE modifications though partially.

### Chronic alcohol exposure affects invasive and migratory capabilities

**Fig. 3:**
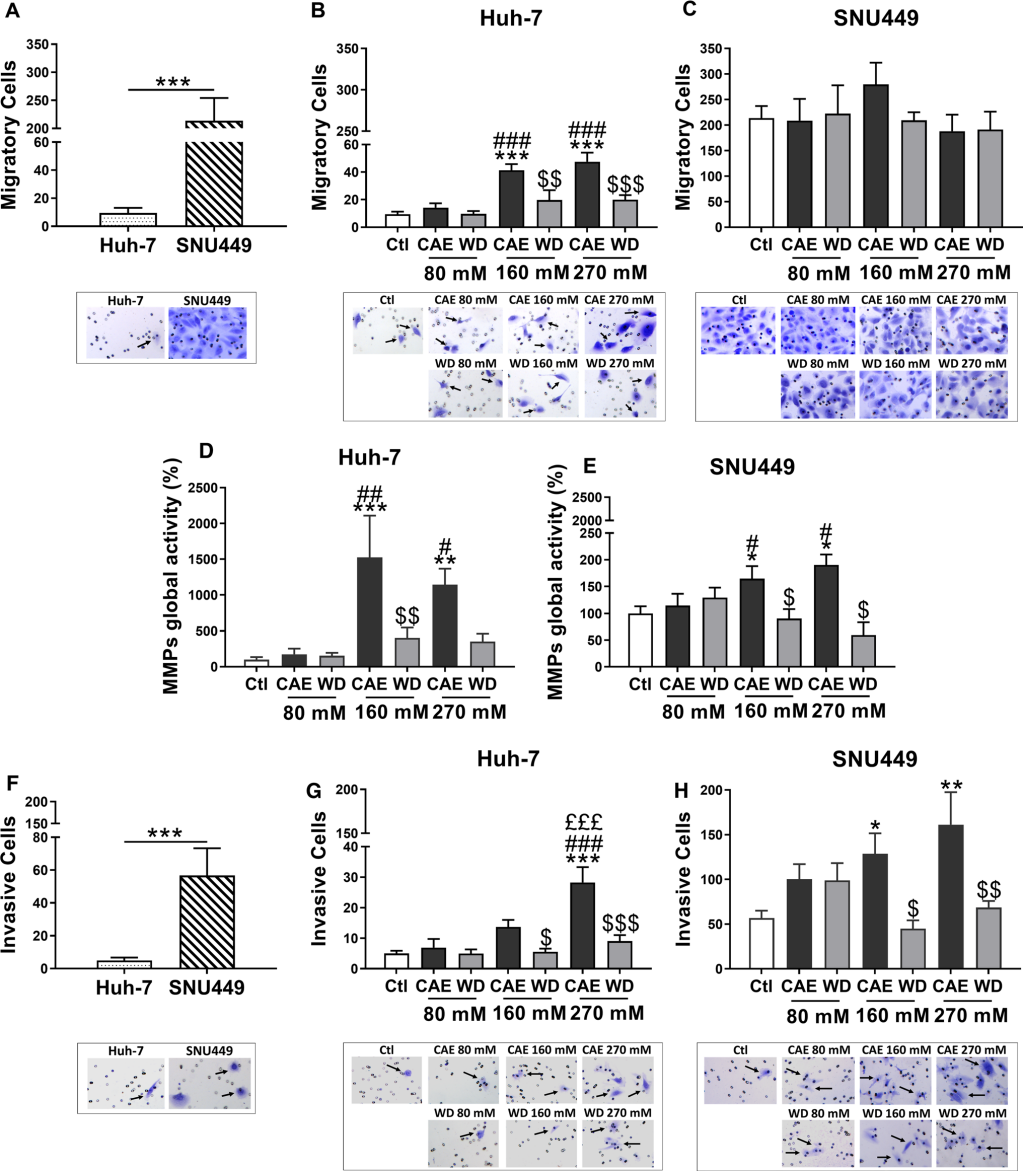
Alcohol withdrawal partially blocked cell aggressiveness induced by CAE. Transwell migration assays were performed in Huh-7 and SNU449 cells. Results represented mean ± SEM of three independent experiments (*N* = 3, ***p < 0.001 vs Ctl, ^###^*p* < 0.001 vs 80 mM, ^$$^*p* < 0.01 vs respective CAE condition, ^$$$^*p* < 0.001 vs respective CAE condition). Photographs of cells stained in crystal violet representing one of the three independent experiment (**A** and **B**). Matrix metalloproteinase global activity was studied in Huh-7 and SNU449 cells. Results represented mean ± SEM of four independent experiments (*N* = 4, *p < 0.05 vs Ctl, **p < 0.01 vs Ctl, ***p < 0.001 vs Ctl, ^#^*p* < 0.05 vs 80 mM, ^##^*p* < 0.01 vs 80 mM, ^$^*p* < 0.05 vs respective CAE condition, ^$$^*p* < 0.01 vs respective CAE condition) (**C **and **D**). Transwell invasion assays were performed in Huh-7 and SNU449 cells. Results represented mean ± SEM of four independent experiments (*N* = 4, *p < 0.05 vs Ctl, **p < 0.01 vs Ctl, ***p < 0.001 vs Ctl, ^###^*p* < 0.001 vs 80 mM, ^£££^*p* < 0.001 vs 160 mM, ^$^*p* < 0.05 vs respective CAE condition, ^$$^*p* < 0.01 vs respective CAE condition ^$$$^*p* < 0.001 vs respective CAE condition). Cells were stained in crystal violet and photographed (**E** and **F**)

Migration and invasion assays were performed to further confirm our earlier observations. Both Huh-7 and SNU449 display cells migration and invasion capabilities. We compared their migratory capability and observed that SNU449 cells presented a higher migration capability than Huh-7 supporting its tumour grades (Fig. 3A). Enhanced migration capability in Huh-7 due to CAE was dose-dependent. Indeed, results showed an increase of 335.62% and 401.05% compared to control for CAE 160 mM and CAE 270 mM, respectively, while only a minimal increase was observed in the CAE 80 mM which was not statistically significant (Fig. 3B). Strikingly, WD significantly restored the migratory phenotype at a comparable level of untreated cells (Fig. 3B). Chronic alcohol exposure and WD did not change the migratory capability of SNU449 cells (Fig. 3C).

Invasive abilities of cancer cell lines correlate highly with their matrix metalloproteinase (MMP) activity. Here, we quantified the global MMP activity of Huh-7 and SNU449 cells to investigate the effect of CAE and WD on their invasive potential. Results of our experiments revealed that CAE induced the MMP activity in Huh-7 cells while WD resulted in reduced MMP activity. The Huh-7 cells exposed to CAE 160 mM displayed an elevated global MMP level by 1526 ± 584.8% while 270 mM CAE resulted in 1144 ± 224.4% increase in comparison to CAE untreated cells. Increase of global MMP levels upon CAE did not follow a dose-response pattern (Fig. 3D). Similarly, global MMP activity in SNU449 cells was also quantified in cells which have undergone CAE and WD. In the case of SNU449 cells, CAE did influence the global MMP levels with significant increase observed only in 160 mM (164.5 ± 23.55%) and 270 mM (190.2 ± 19.66%) CAE while WD totally reversed the effect of CAE and restored the global MMP levels comparable to those of untreated cells (Fig. 3E).

As expected, increased MMP activity results in enhanced invasive capabilities. Mechanistic assays using modified Boyden chamber were performed to assess the invasive capabilities of Huh-7 and SNU449 upon CAE and WD. Since Huh-7 is an early grade and SNU449, an advanced grade cell line, invasion assay was performed to compare the invasive capabilities of both cell lines. Results show that SNU449 cells displayed significantly higher invasive capability in comparison to Huh-7 (Fig. 3F). However, when Huh-7 cells were exposed to our protocol, significant (468.27%) increase in invasive cell number was observed upon 270 mM CAE while WD restored the invasive cell number comparable to control. (Fig. 3G). In the case of SNU449 cells, data represented in Fig. 3H show that CAE significantly increased the invasiveness. While a dose-dependent increase in the number of migrating cells was observed upon 160 mM and 270 mM CAE (Fig. 3H). Strikingly, WD did not show a significant reduction of invasive cells in SNU449 80 mM CAE while invasive cell number was significantly lower in WD after 160 mM CAE and WD after 270 mM CAE (Fig. 3H). The data presented here further emphasized that CAE promoted cancer cell aggressiveness by significantly enhancing the migratory and invasive potential while WD had a beneficial effect with a recovery of almost all parameters.

### Modulation of cancer stem cell markers expression by CAE and WD

**Fig. 4:**
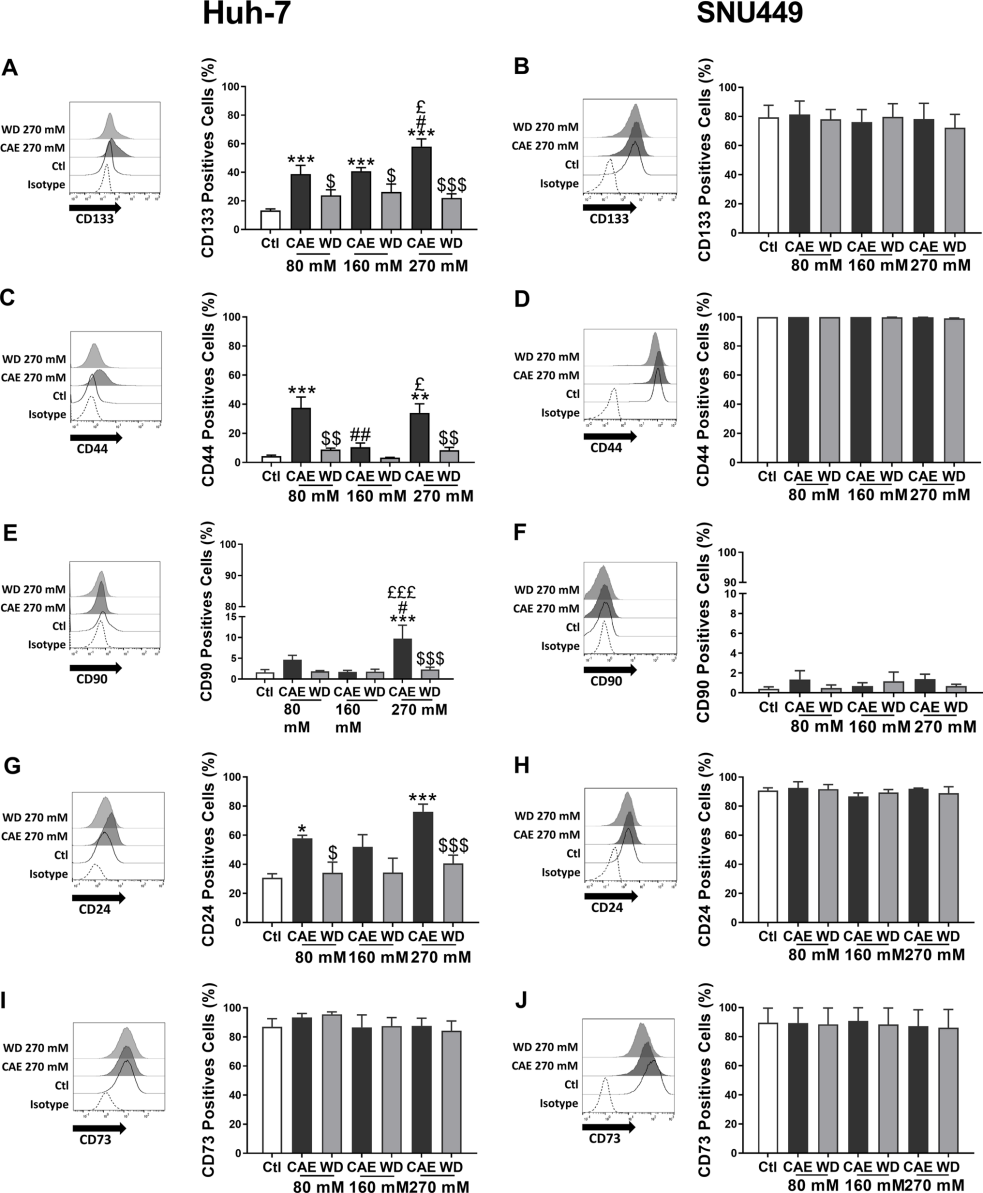
CAE promoted expression of CSC markers in Huh-7 cells, inhibited by withdrawal. Expression of CSC markers CD133 (**A** and **B**), CD44 (**C** and **D**), CD90 (**E** and **F**) was studied using flow cytometry in Huh-7 (**A**, **C**, **E**) and SNU449 models (**B**, **D**, **F**). Results represented mean ± SEM of three independent experiments (*N* = 3, **p < 0.01 vs Ctl, ***p < 0.001 vs Ctl, ^#^*p* < 0.05 vs 80 mM, ^##^*p* < 0.01 vs 80 mM, ^£^*p* < 0.05 vs 160 mM, ^£££^*p* < 0.001 vs 160 mM, ^$^*p* < 0.05 vs respective CAE condition, ^$$^*p* < 0.01 vs respective CAE condition, ^$$$^*p* < 0.001 vs respective CAE condition)

Cancer stem cell (CSC) markers have often been described as markers for tumour aggressiveness and their expression often correlates with poor prognosis. Here, we studied the expression of CD133, CD44, CD90, CD24, and CD73 in Huh-7 and SNU449 cells in the conditions of CAE as well as WD using flow cytometry and RT-qPCR (Suppl. Figure 3 and 4). Flow cytometry analysis of Huh-7 cells revealed that CAE induces the expression of CD133, CD44, CD90, and CD24 while CD73 expression remained unaffected and WD restores the expression levels comparable to those of untreated cells. CAE of Huh-7 cells resulted in increased CD133 positive cell number in a dose-dependent manner. Data presented here show that number of CD133 positive cells increased by 1.9-fold in 80 mM, 2.0-fold in 160 mM, and 3.3-fold in 270 mM CAE. WD partially restored the CD133 positive cell number though not comparable to untreated cells (Fig. 4A). The modulation of CD133 positive cell number after CAE and WD was also reflected in mean fluorescence intensity (Suppl Fig. 5A) suggestive of increased expression. In a similar way, expression of CD133 was also tested in SNU449 cells and observed that CAE and WD did not have a significant effect on the CD133 positive cell number (Fig. 4B) as well as on the mean fluorescence intensity (Suppl Fig. 5B).

CAE and WD also impacted the expression of other CSC marker’s namely CD44, CD90, and CD24 in Huh-7 cells but not in SNU449 cells (Fig. 4). CAE of Huh-7 cells resulted in a 7.6-fold increase of CD44 positive cells at 80 mM while a 6.8-fold increase was seen at the 270 mM dose. CD90 expression was similarly impacted by CAE, 270 mM CAE resulted in five-fold increase of CD90 positive cells. Significant impact on the CD24 positive cell number after CAE was observed in Huh-7 cells while CD73 positive cell number remained unaffected (Fig. 4C, E, G, I). Along with variation in the number of positively stained cells, enhanced MFI suggesting increase of expression was also noted (Suppl Fig. 5C, E, G, I). As observed in the case of CD133, WD partially reversed the effects (Fig. 4C, E, G, I). Interestingly, we observed that in SNU449 cells CAE did not have a profound impact on CD44, CD90, CD24, and CD73 positive cell number (Fig. 4D, F, H, J) as well as on MFI (Suppl Fig. 5D, F, H, J). Results presented here show that CAE has a profound impact on the expression of CSC markers in Huh-7 but not in SNU449 suggesting that Huh-7 cells can acquire characteristics of advanced stage cancer cells under conditions of CAE.

### CAE in Huh-7 cells selectively inhibits GSK3β and activates AKT and ERK signaling pathways

**Fig. 5:**
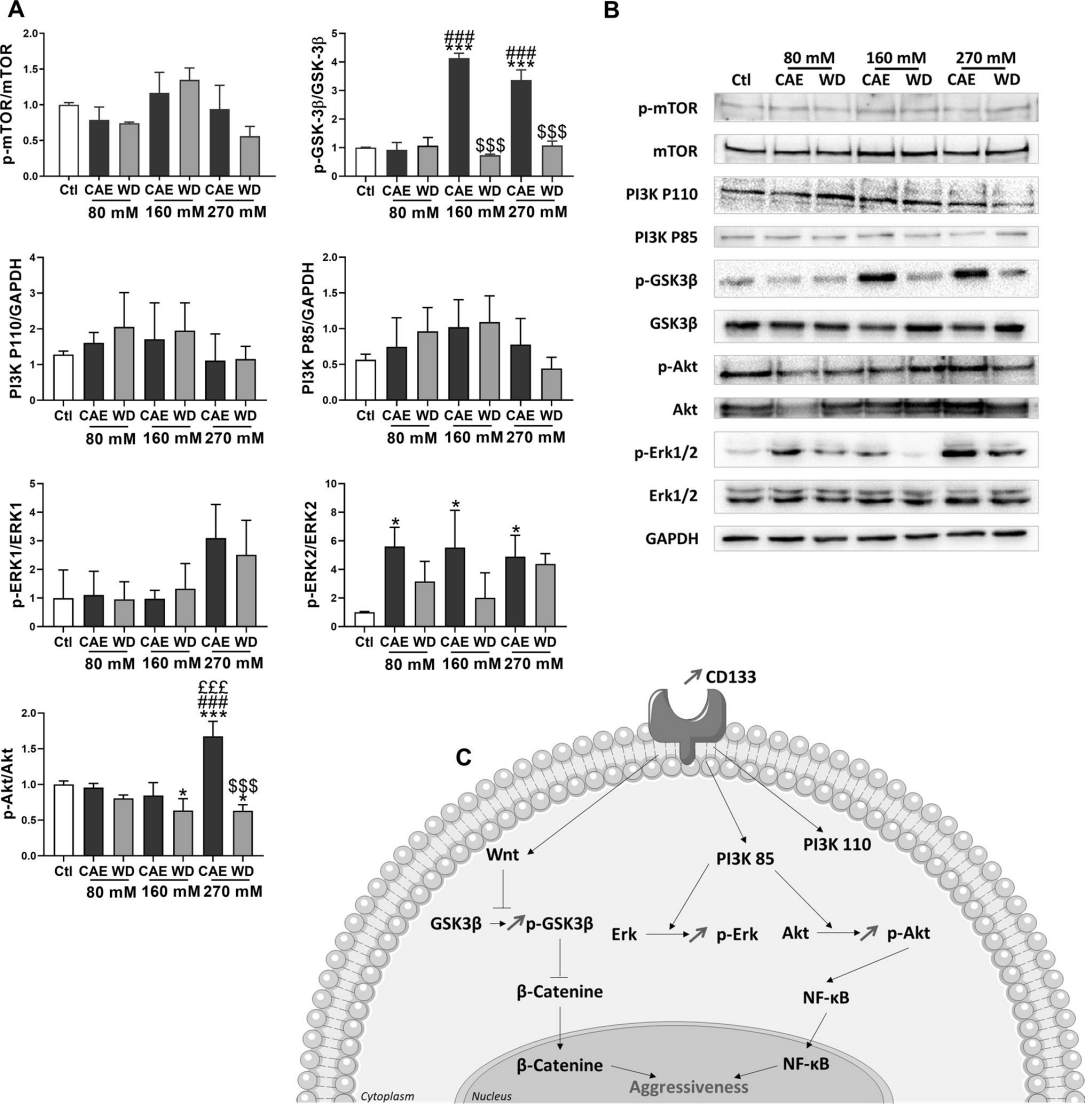
Chronic alcohol exposure modifies signaling pathways in Huh-7. Withdrawal reverses these observations. Protein expression was analyzed by Western Blot in Huh-7 cells. Total extraction was realized. GAPDH, mTOR, GSK3β, Akt, and Erk1/2 were used like controls (*N* = 3) (**A**) Protein quantification were represented mean of activation levels and protein expressions. One of the three independent experiments is presented here (**B**). Results were simplified in schema (**C**)

We investigated the activation of signaling pathways regulating cell invasion with western blot experiments. Densitometry analysis of the immunoblots revealed a 4.141 ± 0.167-fold activation and 3.368 ± 0.355-fold activation of inhibitory site at Ser9 of GSK3β in CAE 160 mM and CAE 270 mM, respectively, compared to control condition. We observed also that CAE 270 mM induced a 1.676 ± 0.120 fold-activation of AKT compared to control condition. Our experiments demonstrated also an increase of ERK 1/2 phosphorylation in CAE conditions (fold-activation of 5.600 ± 0.775, 5.533 ± 1.506, and 4.888 ± 0.750 for CAE 80 mM, CAE 160 mM, and CAE 270 mM, respectively). Very interestingly, WD reversed the phosphorylation modifications induced by CAE. CAE and WD had no effect on mTOR phosphorylation and on PI3K protein levels (Fig. 5A and 5B). Results are schematically summarized in Fig. 5C.

### CD133 expression is higher in HCC patients with alcohol use disorder.

Baseline characteristics of HCC patients are described in Table 1. No clinically relevant differences between groups were observed for median age, sex, BCLC stage, and Child–Pugh liver function class. Concerning biochemical characteristics, our clinical study highlighted a higher rate of alphafoetoprotein, bilirubin, and ALAT in non-alcoholic HCC in compared to alcoholic HCC.

**Table 1:** Baseline characteristics of patients according to HCC etiology

	All etiologies	Alcoholic HCC	Non-alcoholic HCC	Statistic (Alcoholic vs Non-Alcoholic)
Median age (years)	70.96 ± 1.85 (*n* = 67)	70.08 ± 1.412 (*n* = 36)	72.11 ± 2.207 (*n* = 27)	*F* (1.834) = 26.35 *p* = 0.0943
Sex	*n* = 68	*n* = 36	*n* = 28	χ2 Chi-square 0.002546 dl1
Male	88.24% (*n* = 60)	88.89% (*n* = 32)	89.29% (*n* = 25)	*Z* = 0.05048
Female	11.76% (*n* = 8)	11.11% (*n* = 4)	10.71% (*n* = 3)	*P* = 0.9598
BCLC stage	*n* = 64	*n* = 36	*n* = 28	*χ*2 Chi-square 0.005075 dl1
A	10.94% (*n* = 7)	13.89% (*n* = 5)	7.14% (*n* = 2)	*P* = 0.8218
B	12.50% (*n* = 8)	16.67% (*n* = 6)	7.14% (*n* = 2)	
C	25.00% (*n* = 16)	11.11% (*n* = 4)	42.86% (*n* = 12)	
D	4.69% (*n* = 3)	2.78% (*n* = 1)	7.14% (*n* = 2)	
Unkown	46,88% (*n* = 30)	55.56% (*n* = 20)	35.71% (*n* = 10)	
Child–Pugh liver function class	*n* = 64	*n* = 36	*n* = 28	*χ*2 Chi-square 2.055 dl3
A5-6–7	67.19% (*n* = 43)	61.11% (*n* = 22)	75.00% (*n* = 21)	*P* = 0.5610
B7-8–9	12.50% (*n* = 8)	16.67% (*n* = 6)	7.14% (*n* = 2)	
C10-12	6.25% (*n* = 4)	5.56% (*n* = 2)	7.14% (*n* = 2)	
Unkown	14.06% (*n* = 9)	16.67% (*n* = 6)	10.71% (*n* = 3)	
Laboratory values				
Mean alphafoetoprotein (ng/ml)	522.6 ± 339.6 (*n* = 50)	99.38 ± 71.80 (*n* = 28)	1061 ± 760.7 (*n* = 22)	F(88.20) = 21,27 ***p < 0.0001
Mean total bilirubin (μmol/l)	25.56 ± 4.393 (*n* = 32)	30.69 ± 6.638 (*n* = 19)	18.05 ± 4.273 (*n* = 13)	F(3.527) = 18,12 *p = 0.0305
Mean AST (UI/l)	93.78 ± 15.95 (*n* = 32)	79.63 ± 19.12 (*n* = 19)	114.5 ± 27.50 (*n* = 13)	F(1.416) = 12,18 *p* = 0.4900
Mean ALT (UI/l)	72.97 ± 14.57 (*n* = 32)	56.05 ± 10.70 (*n* = 19)	97.69 ± 31.84 (*n* = 13)	F(6.060) = 12,18 ***p = 0.0007
Mean albumin (g/l)	33.98 ± 1.581 (*n* = 30)	33.01 ± 2.178 (*n* = 17)	34.94 ± 2.343 (*n* = 13)	F(1.130) = 16,12 *p* = 0.8449
Mean protothrombine (%)	67.25 ± 3.828 (*n* = 32)	67.63 ± 4.764 (*n* = 19)	66.69 ± 6.587 (*n* = 13)	F(1.308) = 12,18 *p* = 0.5888
Mean creatinine (μmol/l)	89.64 ± 6.802 (*n* = 32)	86.51 ± 8.842 (*n* = 19)	94.22 ± 10.95 (*n* = 13)	F(1.049) = 12,18 *p* = 0.9008

Repartition of sex, BCLC stage, and Child–Pugh liver function class was expressed in percentage. Median age and laboratories values were represented by Mean ± SEM. Data were analyzed by Fisher statistic test.

*p < 0.05

***p < 0.001

**Fig. 6:**
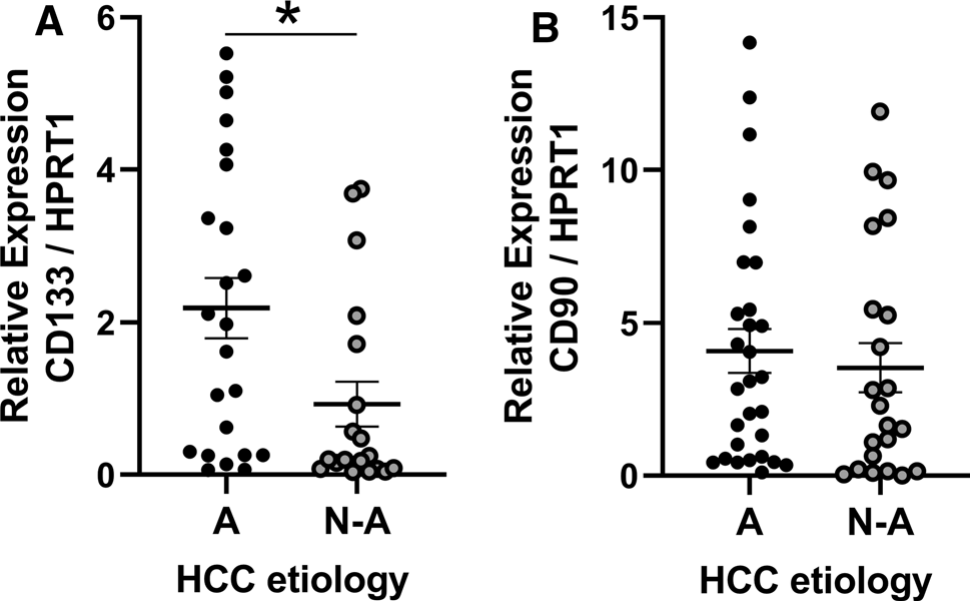
CD133 expression is higher in tumoral tissue of alcoholic (A) HCC compared to non-alcoholic (N-A) HCC. CD133 expression was analyzed by RT-qPCR in tumoral tissue according to HCC etiology (Alcohol: *n* = 23, Others: *n* = 19, *t* test *p* = 0.0179 *p < 0.05) (**A**). CD90 expression was analyzed by RT-qPCR in tumoral tissue according to HCC etiology (Alcohol: *n* = 29, non-alcoholic: *n* = 22, *t* test *p* = 0.6146 not significant) (**B**)

Analysis of CD133 CSC markers by RT-qPCR on tumoural and peri-tumoural samples from HCC patients demonstrated that expression of CD133 is significantly higher in tumour samples in comparison to peri-tumoural samples (Suppl Fig. 6A). We found this same result for the CD90 CSC marker expression (Suppl Fig. 6B). Interestingly, when tumoral samples were segregated based on the etiology of the tumour we observed that CD133 expression was 2.35-fold higher in tumours whose etiology was related to excessive alcohol consumption (Fig. 6A). Segregation according to HCC etiology does not show difference on CD90 CSC marker expression (Fig. 6B).

## Discussion

Here, using two hepatocellular carcinoma cell lines of early and advanced grades, we have demonstrated that chronic alcohol exposure induces aggressive phenotype. We also provided evidence that alcohol withdrawal for a month partially reverses the effect of CAE for most tested parameters.

Product of alcohol metabolism is a well-known causative factor for liver carcinogenesis and progression of HCC [13]. Indeed, 90% of ethanol consumed is metabolized by liver leading to a decrease of its toxicity. However, acetaldehyde, an intermediate compound of ethanol metabolism, is also a well-known human carcinogen [12]. It is the reason why we first investigated ADH and ALDH in our cell lines. While, we found no changes of ADH and ALDH activities or expression following chronic ethanol exposure, we observed basal differences between Huh-7 and SNU449 cell lines. Indeed, we demonstrated reduced activity of ADH and ALDH positive cells in SNU449 compared to Huh-7. Interestingly, our results present an increase of ALDH expression in SNU449 exposed to 270 mM of ethanol which suggests that these cells developed a tolerance/protective mechanism for quick elimination of acetaldehyde. One possible explanation for this variation lies in genetic mutations which modulate both ADH activity and ALDH expression. We are not equal when it comes to metabolizing alcohol and this has an impact on the risk of developing cancer. The ALDH2 2*2 genotype is associated with HCC and is an important risk factor for HCC development. This mutation causes deficiency of ALDH activity leading to an increase in concentration of acetaldehyde and its genotoxic effects following alcohol consumption [31, 32]. Regarding ADH, incidence of HCC is higher in individuals homozygous for ADH1C*1. People with this allele variant produce a more active enzyme leading to acetaldehyde accumulation after alcohol consumption [33]. Genetic variations in ADH and ALDH might explain differences in alcohol metabolism, alcohol toxicity, and aggressiveness between the two cell lines Huh-7 and SNU449.

We report that CAE promoted acquisition of CSCs subpopulation and HCC aggressiveness. Herein, the originality of our protocol is to try to mimic protracted abstinence (1 month) by stopping alcohol treatment after 6 months of prolonged ethanol exposure. Interestingly, alcohol withdrawal inhibits HCC aggressiveness induced by CAE with a decrease of CSC subpopulation. Indeed, withdrawal partially reversed the increase of CD133, CD44, CD90, and CD24 induced by CAE. Thus, the benefit of abstinence in patients with alcohol-attributable HCC described by Costentin et al*.* may be explained by the decrease of cancer cell stemness which is a signal for cancer progression and metastasis [34, 35]. Our results may have relevant implication regarding the previous results of this epidemiological study which described a less overall survival of patients with alcohol-related HCC compared to non-alcohol-related HCC [34, 36]. However, this observation remains debated and an analysis of several studies suggests that the lower overall survival of alcohol-related HCC is not due to an increase of HCC aggressiveness or treatment responsiveness. Indeed, compared to viral HCC, patients displaying excessive alcohol consumption are out of surveillance and diagnosis is delayed [14]. The absence of link between alcohol-attributable HCC and mortality was also observed in breast cancer patients [37].

Cancer stem cells are a small-specific subpopulation characterized by self-renewal and differentiation capabilities and are identified by the expression of distinct cell surface markers such as CD133, CD44, CD90, CD24, and CD73. Huh-7 cells exposed chronically to ethanol expressed CD133, CD44, CD24, CD73, and at fewer level CD90 reflecting the induction of CSCs following ethanol exposure, associated with enhancement of migration and invasion. Our results are corroborated by the fact that CD44 is associated with local aggressiveness and resistance to chemotherapy through regulation of AKT/GSK-3β/β-catenin pathway and epithelial-to-mesenchymal transition genes [38]. CD133 is a CSC marker correlated with poor prognosis in HCC patients, while CD24, another CSC marker, is associated with number and size of tumours and differentiation level of HCC. Moreover, CD24 is also overexpressed in sorafenib-resistant cells [39]. Recovery of low level of CSC markers expression after alcohol withdrawal, observed in our study, may explain the decrease of cell migration and invasion. One of the key cytokines, implicated in the generation of CSCs subpopulation and enhancement of epithelial-to-mesenchymal transition, is TGF-β. TGF-β, induced by ethanol, is an essential regulator of growth and differentiation and has been shown to play a critical role in metabolizing ethanol [40]. All of these observations, demonstrate the critical role of ethanol to interfere in HCC aggressiveness through induction of CSCs subpopulation.

Alcohol abstinence has been suggested to be the gold standard treatment of alcohol-related HCC patients. Epidemiological study showed an increase of overall survival from 7.6 months in non-abstinent alcohol-related HCC patients to 11.7 months in abstinent alcohol-related HCC [34]. In the same time, in chronic alcohol consumer population, risk of upper aerodigestive tract cancer incidence decreases after 10 years of alcohol abstinence to reach the same risk level than non-alcohol population after 20 years of alcohol abstinence [41, 42]. These data suggest the importance of alcohol consumption screening and intervention to reduce and/or prevent alcohol intake to improve not only the overall survival of cancer patients but also their quality of life. Importantly, our results suggest that identification of mechanisms associated with alcohol-related HCC may be useful to develop personalized therapy of patients. Taken together results of earlier studies as well our results suggest that alcohol abstinence can serve as an effective therapeutic strategy towards controlling the progression of HCC in newly diagnosed cases and also slow down invasion in the case of aggressive tumours.

To determine the pathophysiologic impact of abstinence on HCC, we looked at the effect of a 1-month alcohol withdrawal period following a long-term of alcohol exposure of 6 months. This approach is original by two ways. First is the duration with a CAE model that has been extended over 2 months, like it was already developed in breast and colorectal cancers [24, 43]. Second, is by adding a 1-month alcohol withdrawal period. Only a study by Wood *et al*. determined impact of 2-week alcohol withdrawal in a model of CAE in breast cancer [25]. Taken together, our procedure seems to be a good compromise to study the pathophysiological mechanisms of chronic alcohol exposure on HCC associated to the benefits of alcohol management.

In clinical practice, only 0.9% of patients with cancer were informed about the possibility to get help in reducing excessive alcohol consumption [44]. In addition, the few published clinical studies are retrospective. These studies do not provide details on the continuation or cessation of alcohol consumption following the diagnosis, nor on the quantities of alcohol consumed. Consequently, these retrospective data available on alcohol are very fragile because they present many biases. Actually, few specific programs tackle tobacco and alcohol addictions aspect on oncology treatment. Based on self-determination and implementation intention theories and the concept of perceived behavioural control, STAR program in France proposes an innovative way for integrated addiction treatment into the oncology care [45]. This low level of alcohol management asks question about the place of alcohol consumption prevention in the cancer therapy. Indeed, while the impact of alcohol consumption in liver cancer through cirrhosis induction is completely accepted, the question is debated in other cancers for many clinicians. An American study demonstrated that, in 34,080 cancer survey patients, 56.5% were current drinkers, 34.9% exceeded moderate drinking levels, and 21% engaged in binge drinking. This study highlights the prevalence of current alcohol use among cancer survivors, including an increase in alcohol intake over time and higher rates among younger cancer survivors. Among cancer survivors age 18 to 34, 23.6% met the criteria for binge drinking, while only 2.6% of those 75-and-older reported the same [46].

Thus, there is an important gap between the deleterious impact of alcohol use and the opportunity given to the patients to improve cancer prognosis.

In this context, the importance of alcohol consumption screening and prevention in the therapy of cancer must be more deeply explored. Our hypothesis is that cancer diagnosis creates a segregation in patients between a population that adopts a healthy lifestyle with alcohol consumption cessation and another population that adopts an epicurean philosophy of life and continues to drink alcohol.

Our results suggest that alcohol abstinence is an interesting strategy to decrease aggressiveness of HCC cells. The pathophysiological mechanisms of alcohol in HCC progression and aggressiveness remain unclear and additional studies are needed to identify all of the molecular partners and elucidate the critical role of alcohol withdrawal in the management of alcohol-attributable HCC. Our work also brings other questions such as the extent to which alcohol consumption is associated with cancer recurrence and second primary cancer.

## Supplementary Information

Below is the link to the electronic supplementary material.

Supplementary file 1 (TIF 1445 KB)

Supplementary file 2 (TIF 825 KB)

Supplementary file 3 (TIF 1325 KB)

Supplementary file 4 (TIF 1311 KB)

Supplementary file 5 (TIF 2111 KB)

Supplementary file6 (TIF 634 KB)

Supplementary file 7 (TIF 1454 KB)

Supplementary file 8 (TIF 72 KB)

Supplementary file 9 (DOCX 20 KB)

## Data Availability

Enquiries about data availability should be directed to the authors.

## Abbreviations

HCC: Hepatocellular carcinoma
CAE: Chronic alcohol exposure
WD: Withdrawal
HBV: Hepatitis B virus
HCV: Hepatitis C virus
ARLD: Alcohol-related liver disease
CSC: Cancerous stem cell
MMP: Matrix metalloproteinase
ADH: Alcohol dehydrogenase
ALDH: Aldehyde dehydrogenase
T: Tumoral tissue
pT: Peritumoral tissue
A: Alcoholic
N-A: Non-alcoholic
MFI: Mean florescent intensity
