# Supplementary figures and images for: Mechanisms of chronic alcohol exposure-induced aggressiveness in cellular model of HCC and recovery after alcohol withdrawal
Source: Cell Mol Life Sci. 2022 Jun 17;79(7):366. doi: 10.1007/s00018-022-04387-y (PMC9205837; doi:10.1007/s00018-022-04387-y)

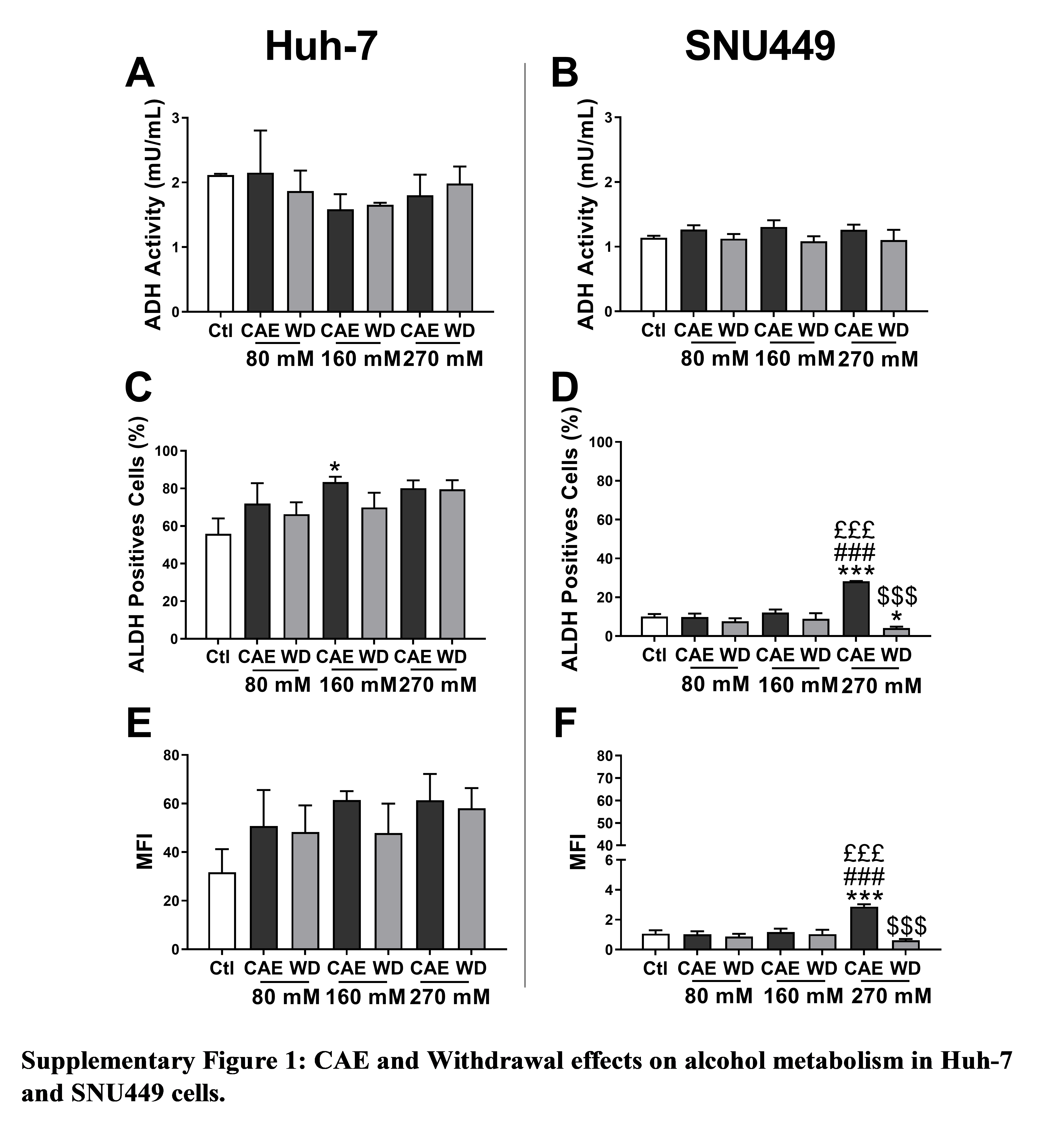

Supplement: Supplementary file 1 — (TIF 1445 KB) [file 18_2022_4387_MOESM1_ESM.tif]

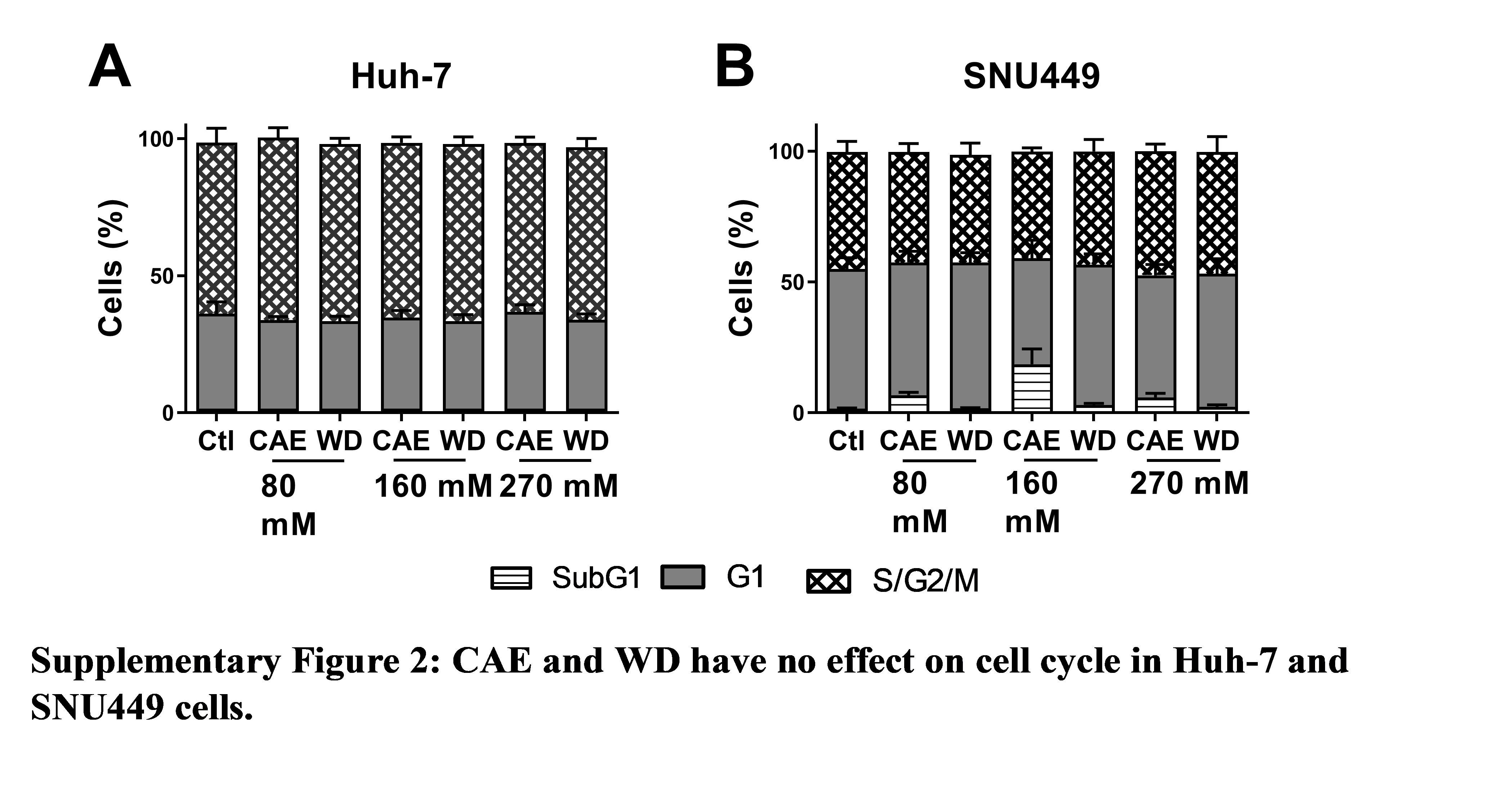

Supplement: Supplementary file 2 — (TIF 825 KB) [file 18_2022_4387_MOESM2_ESM.tif]

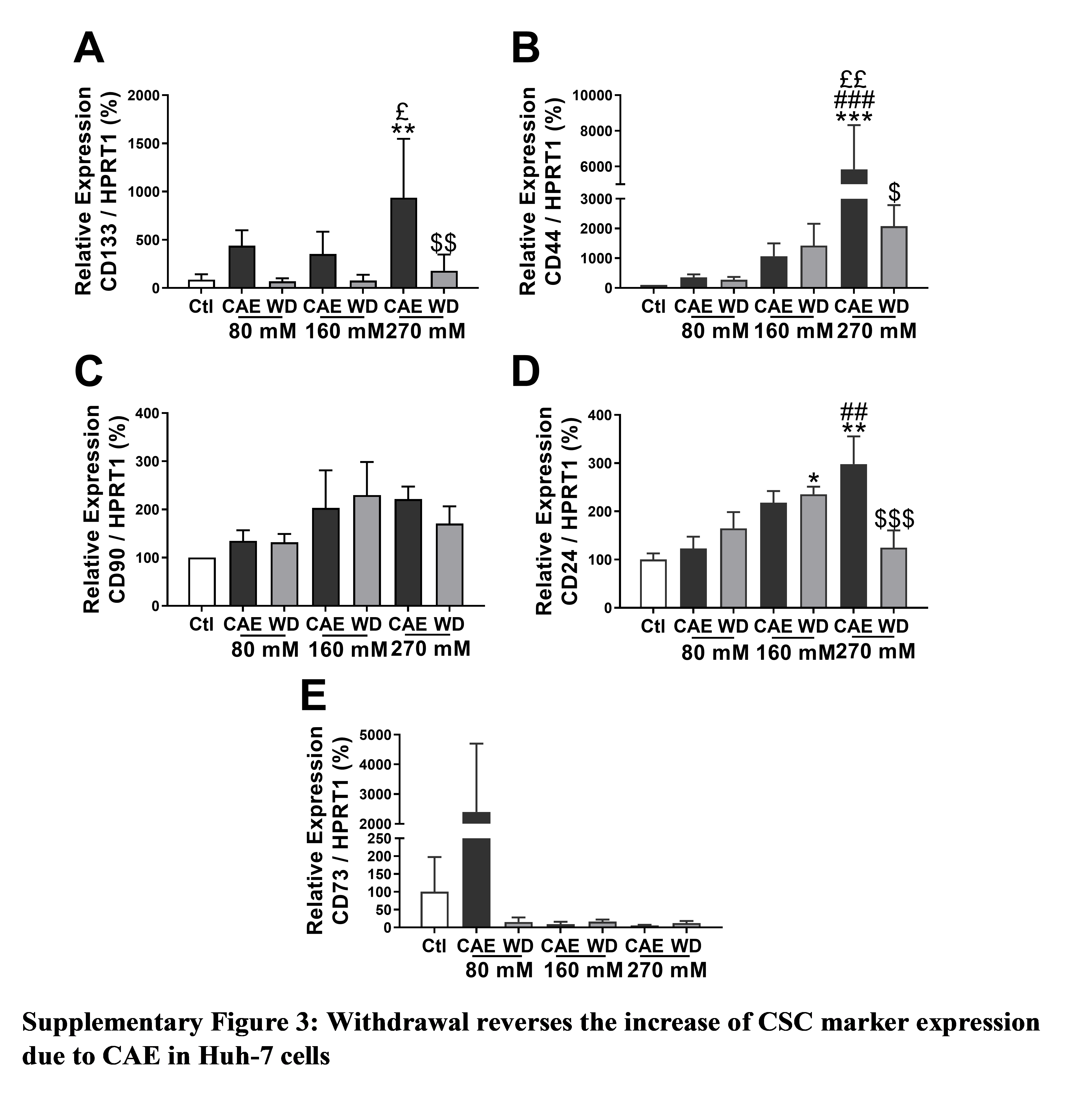

Supplement: Supplementary file 3 — (TIF 1325 KB) [file 18_2022_4387_MOESM3_ESM.tif]

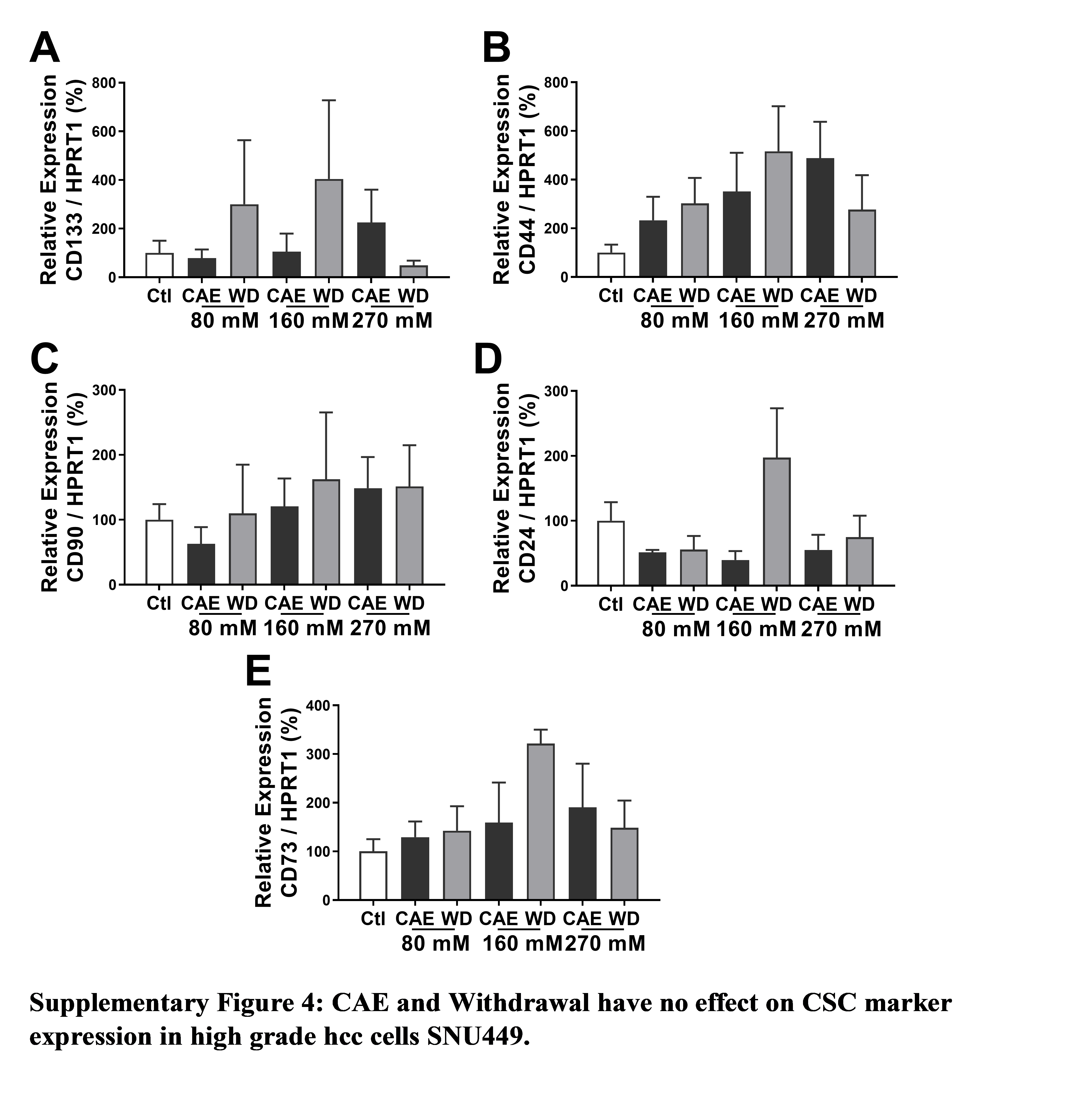

Supplement: Supplementary file 4 — (TIF 1311 KB) [file 18_2022_4387_MOESM4_ESM.tif]

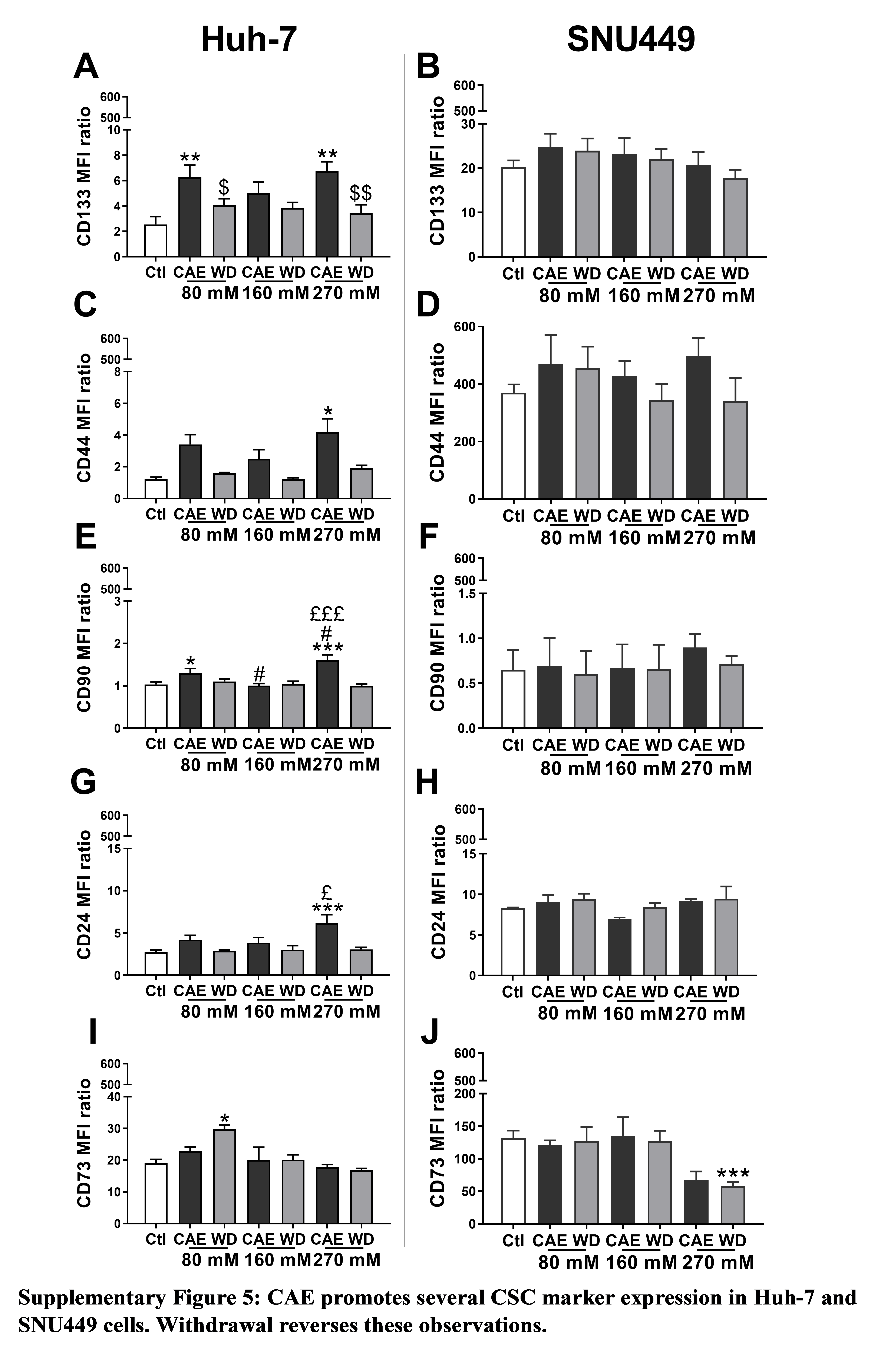

Supplement: Supplementary file 5 — (TIF 2111 KB) [file 18_2022_4387_MOESM5_ESM.tif]

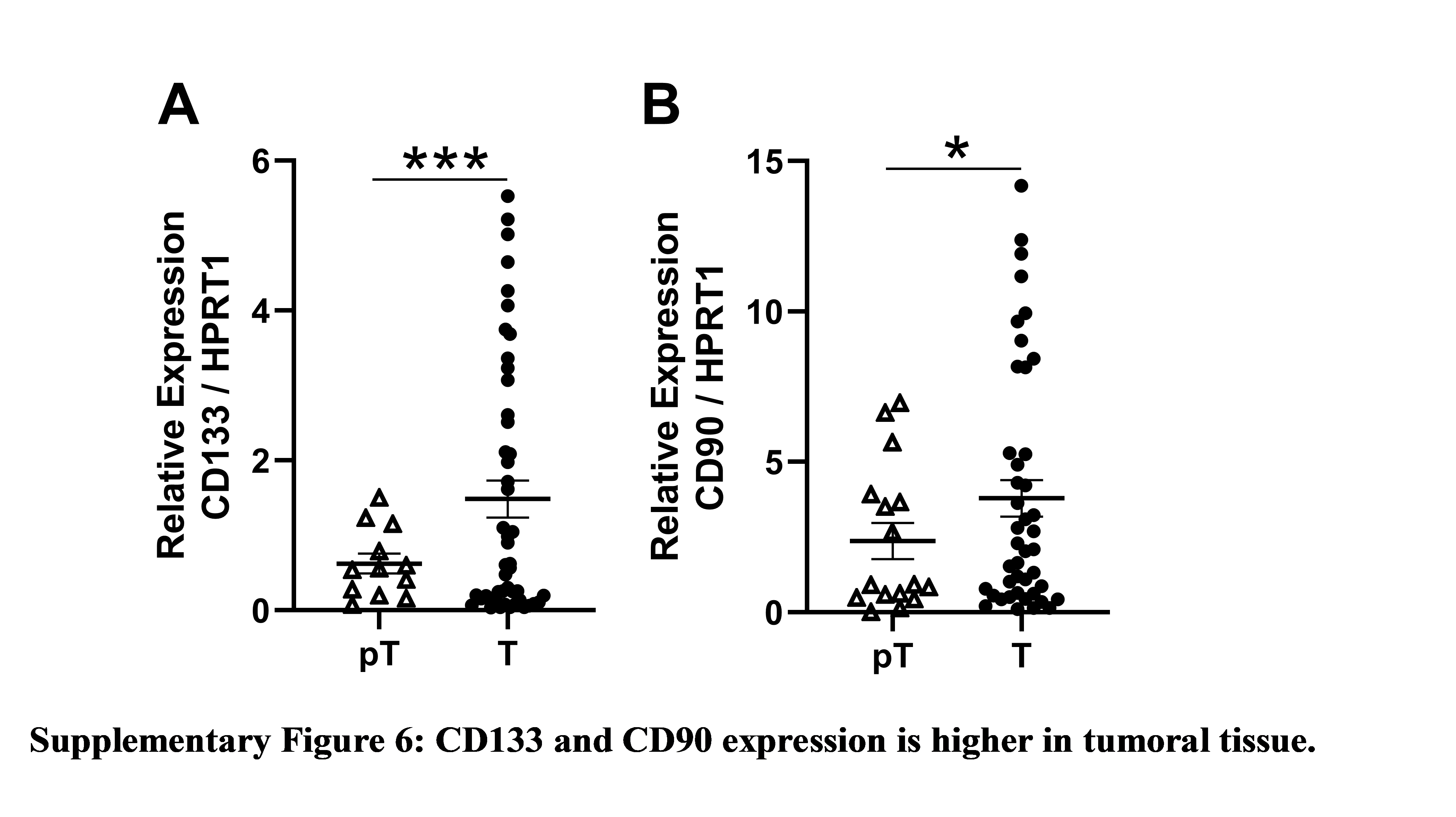

Supplement: Supplementary file 6 — Supplementary file6 (TIF 634 KB) [file 18_2022_4387_MOESM6_ESM.tif]

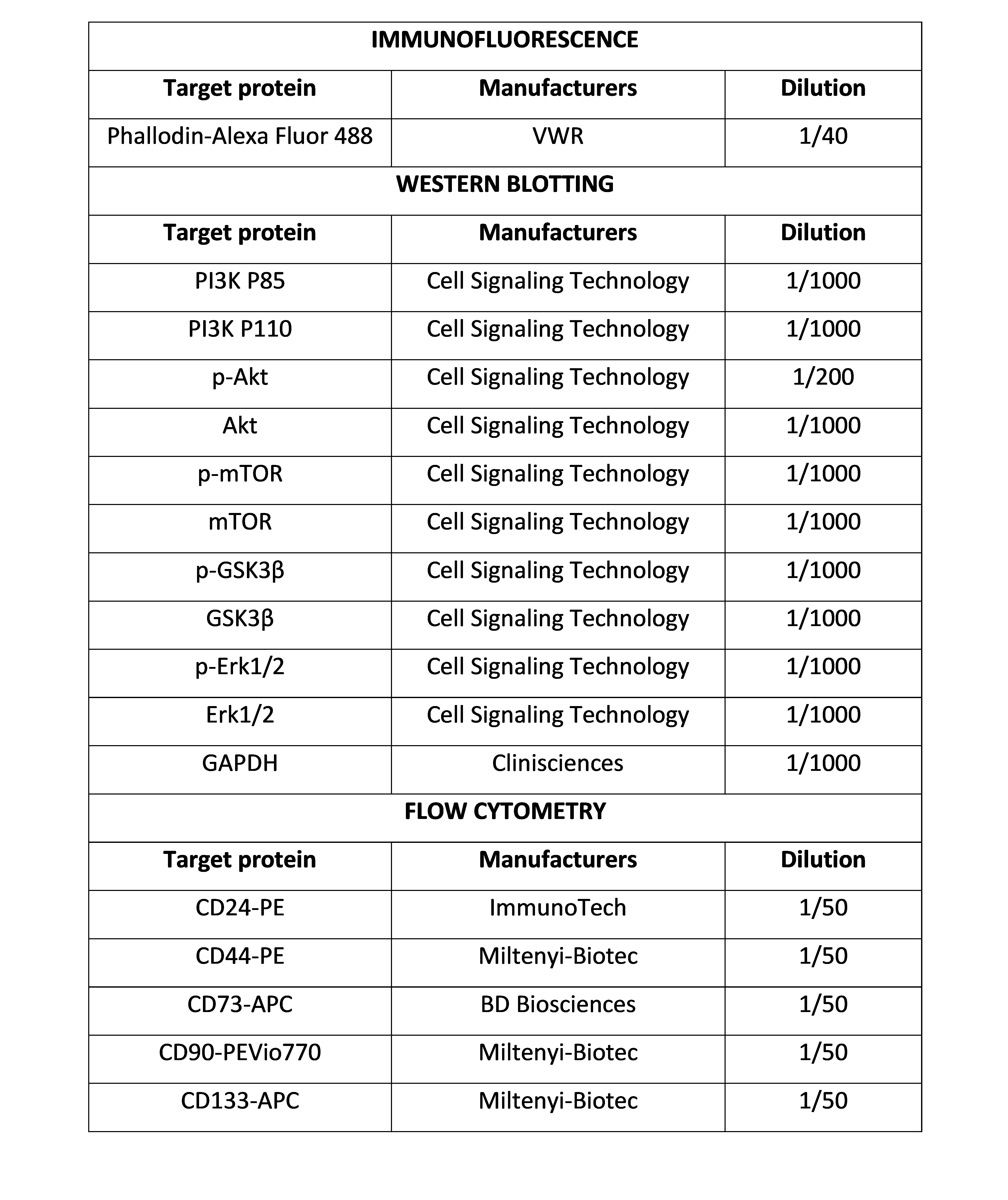

Supplement: Supplementary file 7 — (TIF 1454 KB) [file 18_2022_4387_MOESM7_ESM.tif]

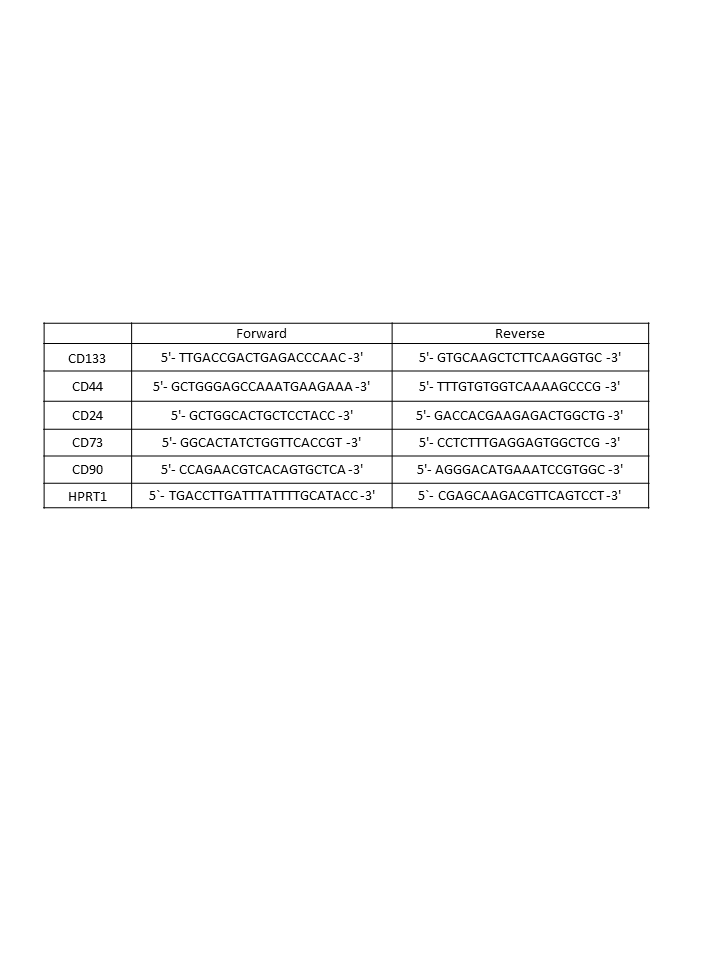

Supplement: Supplementary file 8 — (TIF 72 KB) [file 18_2022_4387_MOESM8_ESM.tif]
