# Supplementary material for: Mechanisms of chronic alcohol exposure-induced aggressiveness in cellular model of HCC and recovery after alcohol withdrawal
Source: Cell Mol Life Sci. 2022 Jun 17;79(7):366. doi: 10.1007/s00018-022-04387-y (PMC9205837; doi:10.1007/s00018-022-04387-y)
Supplement: Supplementary file 9 — (DOCX 20 KB) [file 18_2022_4387_MOESM9_ESM.docx]

**Supplementary Materials and Methods**

**Alcohol Dehydrogenase assays**

Alcohol dehydrogenase (ADH) assay was performed using Alcohol dehydrogenase activity kit according to manufacturer’s instructions (Sigma-Aldrich-Merck, Darmstadt, Germany). Assay was always performed in duplicate. Absorbance at 450nm was measured using Tecan Infinte M200 Pro. The ADH activity was determined by the following equation :

$$ADH activity= \frac{B x Sample Dilution factor}{\left( Reaction Time \right)x V}$$

With

- B : the amount (nmole) of NADH generated between T_initial_ and T_final_
- Reaction time : T_final_ - T_initial_  (in minutes)
- V : sample volume (in mL) added to well

**Aldehyde Dehydrogenase Assays**

Aldehyde dehydrogenase was assayed using Aldefluor kit (StemCell Technology, Vancouver, Canada) following manufacturer’s instructions. Cells undergone CAE and WD were stained and analysed in flow cytometer (MACSQuant cytometer running MACSQuantify software; Miltenyi Biotec, Paris, France).

**Cell Cycle analysis**

For cell cycle analysis, cells were removed using a sterile scraper and washed twice with PBS. Ethanol at 70% concentration was added to the cells and left at -20°C overnight. Alcohol was removed and cells were incubated in PBS containing 0.1% Triton X100 for 5 min. Cells were then incubated in 15µg/ml of propidium iodide and 100µg/ml of RNAse and analysed in flow cytometer. The sofware Miltenyi Biotec determined automatically the different stages of cell cycle.

**Cell Viability assays**

The methylthiazole tetrazolium (MTT) salt assay was performed to determine the impact of CAE and WD on cell viability. At 24h, 48h, 72h and 96h after seeding, cells were treated with 0.5 mg/ml MTT in culture medium for 1 hr. After incubation, formazan crystals were dissolved with DMSO:Isopropanol (50:50). Absorbance at 570 nm was measured by using Tecan Infinite M200 Pro.

**Flow cytometry**

Flow cytometry analysis was used to determine the impact of CAE and WD on expression of cancerous stem cells (CSC) markers. Cells were incubated in a saturation solution PBS and 2.5% mouse serum for 30 minutes at 4°C. Cells were stained with fluorescent-conjugated primary or isotype-matched antibodies for 30 minutes at 4°C. Cells were washed twice with PBS and analysed in a flow cytometer and data for 100,000 events was collected. Results were expressed in percentage of positive cells and mean fluorescent intensity (MFI) ratio. Results were analysed using FlowJo software.

**Immunofluorescence assays**

To determine morphology cell and circularity index, cells were washed three times with PBS. Cells were fixed with 4% paraformaldehyde at 4°C for 15 minutes. Cells were then washed with PBS and permeabilized with PBS/0.5% Triton X100 for 10 minutes. After PBS wash, cells were stained with Alexafluor 488-conjugated phalloïdin at a dilution 1:40 for 20 minutes at room temperature. Later, cells were washed with PBS and incubated with 50µg/mL of DAPI for 15 minutes. Cells were washed with PBS and mounted on microscopic slide. Cells were observed in a fluorescence microscope and photographed. Results were analyzed using ImageJ (reference/citation)

**Matrix MetalloProteinases (MMP) ’s global activity assays**

Global MMP’s in cells undergone CAE and WD were quantified using MMP activity assay kit (Abcam) following manufacturer’s instruction. Briefly, 10 µl of cell supernatant was collected and placed in a 96 well-plate and 10µl d’APMA (4-Aminophenylmercuric Acetate) at final concentration of 1mM was added. An incubation of 2 hours at 37°C is necessary to observe MMP-2 (gelatinase), MMP-7 (matrilysin), MMP-8 (neutrophil collagenase), MMP-9 (gelatinase), MMP-11 (stromelysin-3), MMP-12 (macrophage elastase), MMP-13 (collagenase-3) and MMP-14 activity. After this incubation, 20 µL of MMP Green Substrate working solution (dilution 1:100) was added. Samples were gently mix and incubated at room temperature in dark. Flourescence at 525nm was measured using Tecan Infinite M200 Pro. **Migration and invasion assays**

For the migration assay, cells were seeded in 200µl of medium on the top of Boyden Chamber (BD Biosciences, 24-wells plates, 8µm pore size). Bottom chamber contained 800µl medium. After 24h, cells were washed twice with PBS and fixed using methanol for 10 min. Alcohol was removed and cells were stained with crystal violet for 5 min followed by water wash three times. Migrated cells were observed in an inverted microscope and counted in 20 separate fields. These assays were performed in triplicate.

For the invasion assays, Boyden chamber were coated with matrigel (BD Biosciences, 24-wells plates, 8µm pore size). The same protocol was described previously for migration assay was used.

**Mortality analysis**

To analyse mortality in cells undergone CAE and WD, cells were suspended in PBS containing 40ng/ml propidium iodide. Stained cells characteristic were directly collected by flow cytometry and analysed using FlowJo software.

**RNA extraction, quantitative real time PCR**

Total RNA was extracted using ReliaPrep™ RNA Miniprep Systems (Promega, Charbonnières-les-bains, France). After RNA quantification, 1 µg of total RNA was used to prepare cDNA using Reverse transcriptase kit (Applied Biosystems by Thermo Fisher Scientific Quantitativ. Real time PCR was performed using a SYBR probe. Sequence of primers used for quantitative Real Time PCR (RTqPCR) are presented in Supplementary table 2. HPRT1 gene was used as reference gene.

**Western Blot analysis**

To extract total protein, cells were lysed with Nonidet P40 (NP40) buffer containing protease and phosphatase inhibitors (Roche, Meylan, France) and Triton-X100 at finally concentration of 0.1%. After protein quantitation (Bradford), equal amounts of each protein sample were separated by electrophoresis and transferred on PVDF membranes (Bio-Rad, Munich, Germany). Primary antibodies using are listed in Supplementary table 1. After secondary antibody incubation, Blot’s were developed using Enhanced ChemiLuminescence (ECL) system (Bio-Rad, Munich, Germany). Results were analysed using ImageLab software.
